# Distributed Health Literacy Among People With Intellectual Disability, Their Supporters and Healthcare Professionals: A Scoping Review

**DOI:** 10.1111/hex.70548

**Published:** 2026-03-18

**Authors:** Maryann Barrington, Karen R. Fisher, Ben Harris‐Roxas, Julian N. Trollor, Catherine Spooner, Julie M. Christensen, Janelle Weise

**Affiliations:** ^1^ National Centre of Excellence in Intellectual Disability Health UNSW Sydney Sydney Australia; ^2^ Social Research Policy Centre UNSW Sydney Sydney Australia; ^3^ School of Population Health UNSW Sydney Sydney Australia; ^4^ International Centre for Future Health Systems UNSW Sydney Sydney Australia; ^5^ Faculty of Medicine and Health University of Sydney Sydney Australia

**Keywords:** decision‐making, health information, health literacy, health promotion, intellectual disability

## Abstract

**Background:**

Health literacy is associated with improved healthcare experiences and health outcomes and is influenced by the social context in which it occurs. People with intellectual disability face stark health inequalities, yet the health literacy concept is underexplored for this group. Little is known about how health literacy is co‐constructed between people with intellectual disability, supporters and healthcare professionals.

**Objective:**

The aim is to understand the experiences of people with intellectual disability accessing, understanding, appraising and applying health information together with their supporters and healthcare professionals.

**Search Strategy:**

This scoping review followed Joanna Briggs Institute guidelines. Articles were identified and retrieved from CINAHL, PsycINFO, PubMed and EMBASE. Articles were included if they were published between 2000 and the present and focussed on aspects of how people with intellectual disability accessed, understood, appraised or used information or the role that socio‐environmental influences, including support networks and healthcare professionals, have in this process.

**Data Extraction and Synthesis:**

Two reviewers completed abstract and full‐text screening, addressing any conflicts at each stage. Data were extracted and coded deductively, according to the integrated model of health literacy.

**Main Result:**

Following search and screening, 90 articles were included for review. Interpretation of the evidence suggests that health literacy is a relational process between people with intellectual disability, support networks and healthcare professionals. Each group experiences particular barriers and facilitators to this process and is impacted by its wider social and environmental contexts. There was limited evidence about how personal characteristics might shape health literacy, particularly intersectional experiences.

**Discussion and Conclusions:**

Health literacy is a social practice, with roles and responsibilities shared among people and systems. Healthcare and disability sectors can facilitate health literacy by creating environments that support shared access and use of health information, as well as facilitate choice and decision‐making.

**Patient or Public Contribution:**

Collaboration with people with intellectual disability in scoping reviews is an emerging area. We gained the perspectives and feedback of colleagues with lived experience of intellectual disability for the design of the review and interpretation of the evidence. This included meeting with a Lived Experience Reference Group of seven people with intellectual disability to discuss our process and findings and receive their guidance. Their contributions supported how we interpreted the findings and reported the review.

## 1 Introduction

The United Nations Convention on the Rights of Persons with Disabilities (UNCRPD) recognised the right of people with disability to attain the highest standard of healthcare without discrimination [1]. Nonetheless, people with intellectual disability experience stark health inequalities. This includes living on average 27 years less than the general population [2, 3] and having a higher prevalence of health conditions such as epilepsy, sensory, metabolic and nutritional disorders [4]. Higher health literacy may support patient empowerment as it fosters a greater understanding of health and health systems and increases the capacity to be involved in decision‐making [5]. However, people with intellectual disability are under‐represented in the health literacy literature, which makes efforts to explore the implications of the construct for this population an important step towards addressing health inequalities [6].

**Figure 1:**
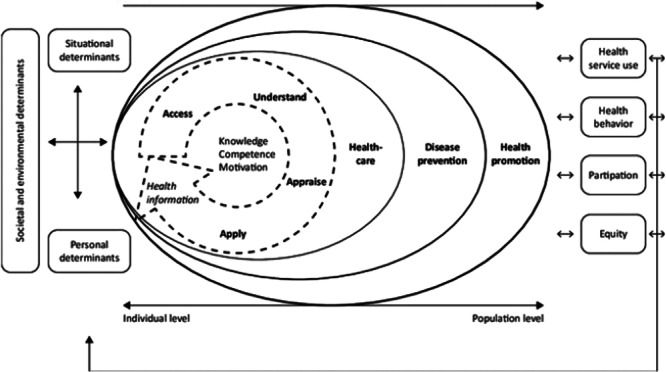
Integrated model of health literacy. *Note*: This model is from Sørensen et al.'s [10] scoping review of health literacy definitions and models.

Definitions and frameworks of health literacy range from functional processes (e.g., reading and writing skills) to critical approaches addressing social determinants of health [7, 8]. Sørensen et al.'s widely cited integrated model defined it as how people access, understand, appraise and apply health information to make decisions [9] (Figure 1). The model also acknowledges that contexts and environments may support or impede the access and use of health information [11, 12, 13]. This integrated model has shown utility for understanding experiences of underserved groups, including rural [14] and socio‐economically disadvantaged communities [15]. It has also been used to design health literacy interventions for people with disability [16]. Despite the model's inclusion of social and environmental influences, measures and interventions continue to defer to person‐level skills and responsibilities [16, 17], implying a disconnect between theory and application. This disconnect highlights a need to refine existing models to better reflect relational and distributed aspects of health literacy, particularly for people with intellectual disability.

The organisational model of health literacy more explicitly redirects the focus away from the person towards system‐wide relational constructs [11, 18]. This model is used to explore the role organisations have in providing access to health information, supporting service users to understand and use information, and facilitating informed consent and decision‐making. Similarly, distributed health literacy orients it as a social practice determined by the context in which it occurs [19, 20]. These models support a focus towards socio‐environmental resources and actors (e.g., families and schools) and how they influence the practice of health literacy [20].

Available research on health literacy for people with intellectual disability typically focusses on knowledge retention for specific topics (e.g., nutritional information) [21, 22, 23]. Socio‐environmental processes such as historic policies of institutionalisation, which saw people with intellectual disability placed in state‐run institutions separated from mainstream society, have ongoing impacts on access to health information [24]. Institutionalisation left a legacy of ableist attitudes, whereby people with intellectual disability continue to be viewed as passive recipients of healthcare, who do not want or need health information, or who cannot or do not use health information to make decisions about their own health [7, 25, 26].

Prior reviews of health literacy among this population highlight that power imbalances, environmental factors and available supports influence health interactions and therefore health literacy [7, 26, 27]. For example, a review of *communicative* health literacy interventions for people with intellectual disability found that it was not limited to individual activities but occurred within a social context [26]. However, these reviews have tended to emphasise the measurement of outcomes and the efficacy of individual‐level interventions, without fully exploring the broader social processes, environmental conditions and distributed responsibilities that shape how health literacy is practised in everyday life [28]. This scoping review addresses that gap by moving beyond person‐level accounts to examine health literacy as a relational and socially embedded process [11, 29].

## 2 Methods

### 2.1 Approach and Research Questions

Health literacy is an emerging and developing concept, with roots in patient education, health information and health promotion. We selected a scoping review because they are suitable for exploring and summarising emerging research areas, particularly where diverse study designs and evidence will be included. This scoping review was conducted in accordance with the Joanna Briggs Institute (JBI) methodology for scoping reviews. Reporting followed the Preferred Reporting Items for Systematic Reviews and Meta‐Analyses Extension for Scoping Reviews (PRISMA‐ScR) [30]. A registered protocol was published on Open Science Framework (URL: https://osf.io/uyn5h).

The final wording of our review questions slightly deviates from the registered protocol. During screening and data extraction, it became clear that the literature often described the process of accessing, understanding, appraising and applying health information without explicitly using the term health literacy. To ensure comprehensiveness, we clarified the review questions to capture both perspectives while maintaining alignment with the protocol's intent. These refinements did not alter the scope of the review but provided greater precision in reporting how evidence was conceptualised in the literature.

The research team, including team members with intellectual disability and a Lived Experience Reference Group (LERG) involving seven people with intellectual disability, planned the review using both the health literacy model and their knowledge and lived experiences of health information accessibility. The LERG identified what the key aims and contributions of the review should be and preliminarily proposed the key elements that may come through the final review (e.g., health literacy as a social activity). On the basis of this information, we identified the primary research question guiding this review as *what is known about the factors that support or impede health literacy for people with intellectual disability?* The secondary question asked *what are the roles and experiences of supporters and HCPs in this process?*

### 2.2 Search Strategy

An initial limited search of EMBASE was undertaken to identify exemplar articles on the topic. The words contained in the titles and abstracts of relevant articles and the index terms used to describe the articles were compiled to develop a full search strategy for EMBASE, PsycINFO, PubMed and CINAHL (see Figure S1). Keywords were selected based on how frequently they appeared in exemplar articles identified in the initial search. These words were combined using Boolean operators and adapted to the indexing terms used in each database (e.g., MeSH in PubMed and Thesaurus terms in PsycINFO). For example, in PubMed, the MeSH term “Intellectual Disability” was combined with “Health Literacy” OR “Health Education,” while in PsycINFO we used the subject headings “Learning Disorders” AND “Health Knowledge, Attitudes, Practices”.

The search strategy was discussed with an academic librarian and with a working group of researchers with expertise in public health and intellectual disability health. As health literacy is strongly related to health promotion and health education, research from these related areas was also searched and considered in this review. All databases were searched on 12 July 2024. Reference lists of all included sources of evidence were screened for additional studies, as were the reference lists of published reviews on related topics. Grey literature was identified through Google Scholar and Google Advanced searches. Only studies published in English, between 2000 and the present, were included.

### 2.3 Screening

Sources returned in the search were uploaded to EndNote, and duplicates were removed. The remaining sources were then uploaded to Covidence for screening. Two independent reviewers performed title and abstract screening using predefined inclusion and exclusion criteria (Table 1). The same two reviewers then completed full‐text screening. Discrepancies at each stage were resolved through discussion, and if consensus was not reached, a third senior team member was available to adjudicate, but this was not required. To promote consistency, the team piloted the criteria on a sample of 20 abstracts prior to full screening, refining definitions and clarifying decision rules.

**Table 1:** Inclusion and exclusion criteria.

Component	Inclusion	Exclusion
Population	People with intellectual disability, their supporters and HCPs Included children and adults from any nation	Studies that combined intellectual disability with neurodevelopmental or cognitive disability, or other disability and did not stratify results by group
Concept	The development of health literacy. Health literacy was defined using both capability frameworks and Sørensen et al.'s integrated model Health literacy: how people access, appraise, understand and use health information (written, visual or verbal) and how they are supported to do so Environmental capabilities that act as barriers and facilitators to this process were of interest (e.g., policies, resources, the health literacy of supporters and psychosocial factors)	Studies that focussed on measuring health information or health outcomes after an intervention without considering the processes for how these outcomes occurred or the role that health literacy or health information may have played in this outcome
Context	All contexts where health information was accessed, either formally or informally and where health decisions were made	
Sources	Qualitative and quantitative studies. Original studies, discussion papers and book chapters	Conference posters and abstracts, textbooks, protocols and reviews

Given that we used search terms beyond health literacy (e.g., health promotion and health education), many conflicts related to sources which bordered between a health literacy and a non‐health literacy focus. The team decided that articles that explored how people with intellectual disability accessed, understood, appraised or used health information—or the role of supporters and HCPs in the process—would be considered articles about health literacy. For example, health promotion articles which focussed on marketing health information and enhancing community understanding were considered for inclusion. Health promotion articles which focussed on intervention uptake without reference to health information and understanding were not considered for inclusion.

We included original empirical studies as well as non‐empirical sources such as book chapters, discussion papers and commentaries. This decision was consistent with scoping review methodology, which seeks to map the breadth of available evidence rather than restrict inclusion to particular study designs. Non‐empirical sources were valuable for identifying conceptual framings, theoretical debates and practice‐based insights in an area where empirical research remains limited.

Although children and adults with intellectual disability can have different health literacy experiences and support needs, we included both groups to map the breadth of available evidence, consistent with the exploratory nature of scoping reviews.

### 2.4 Data Extraction and Synthesis

Data were extracted independently by two reviewers using a data extraction tool created in Excel. One reviewer extracted from all included full‐texts, and the other reviewer extracted from half of the included full‐texts. This allowed checking consistency and coverage in the approach to extraction while also fitting within the available human resources of the team. Consistency was assessed by comparing extracted data across the overlapping sample, and any discrepancies were discussed and resolved collaboratively. The extraction template was iteratively refined during this process to ensure that variables were applied consistently across studies.

The extraction tool charted methodological and study design information for each source. Deductive and inductive methods of data extraction and charting were used to answer the research question. Deductive extraction was guided by the key components of the integrated model. This included data related to how people access, understand, appraise and use information and data relating to the role of personal, social and environmental and situational determinants. Inductive analysis involved noting data which did not easily fit into this conceptualisation in a final column of the extraction tool. Both methods were employed to facilitate an iterative and comprehensive understanding of the literature, including capturing any key concepts or processes which did not align neatly with the integrated model [31].

Empirical sources were prioritised for identifying patterns of lived experience and practice, while non‐empirical sources were integrated to contextualise these findings, illuminate gaps in evidence and highlight theoretical or policy implications. For example, discussion papers were particularly important in clarifying how distributed health literacy might apply to people with intellectual disability, even where empirical testing was absent. This approach enabled us to situate empirical findings within wider conceptual and policy debates, strengthening the interpretation of themes.

Once key themes had been identified from extracted data, these themes were shared with the LERG. The LERG provided feedback about how relevant the themes were to their experiences and the implications our themes have for policy and practice. The LERG further supported us in refining our themes. For example, where we originally identified ‘supported decision‐making’ as a theme from the data, the LERG encouraged us to return to the literature and identify whether there was evidence for independent decision‐making, as they felt this to be an important addition. Upon return, we identified evidence to build and expand this theme and capture what was important from a lived experience perspective.

### 2.5 Quality Review

While not a requirement of a scoping review, a quality assessment of the literature was conducted using Hawker et al.'s [32] critical appraisal tool for assessing studies from different paradigms. This tool involved scoring publications according to nine quality criteria using 4‐point scales ranging from *1—Very poor* to *4—Good*. These nine criteria assessed the quality of a publication's abstract, introduction, method, sampling, analysis, reporting of ethics and bias, reporting of results, generalisability and reporting of implications. Scores between 9 and 23 were defined as ‘poor’, between 24 and 28 ‘moderate’ and those between 29 and 36 ‘good’. Consistent with scoping review methodology, studies were not excluded or weighted on the basis of quality. Instead, they provided context for interpreting confidence in our findings.

## 3 Results

### 3.1 Article Characteristics

**Figure 2:**
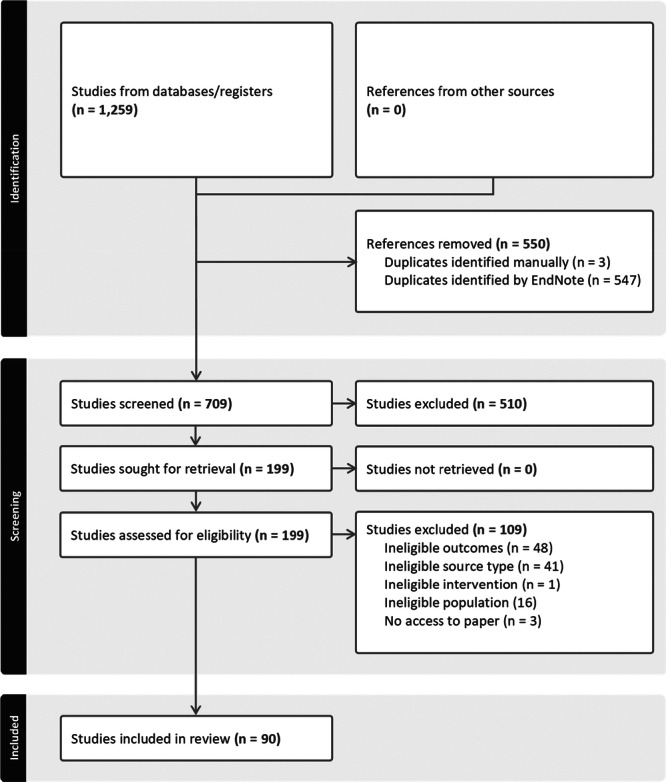
PRISMA search results.

The search returned 1259 publications (see Figure 2). After removing duplicates and conference abstracts and undertaking title and abstract and full‐text screening, 90 publications were included in the review (see Table S1). Of these, 77 publications were empirical (55 qualitative, 9 quantitative and 13 mixed methods) and 13 were literature reviews, commentaries or discussion papers.

Sources were from high‐ and middle‐income countries, with most from the United Kingdom (*n* = 26), the United States (*n* = 12) and Australia (*n* = 11). Participants from included sources comprised people with intellectual disability (*n* = 60), formal and informal supporters (*n* = 47) and healthcare providers (*n* = 16).

### 3.2 Quality Assessment

The average score for the 77 included empirical studies was 27, with a range of 18–34. Most studies were within the middle range of quality, with 19 studies graded as ‘good’, 48 graded as ‘moderate’ and 23 as ‘poor’ quality. Altogether, the quality appraisal outcomes suggest that sampling was not fully reported or justified, ethics and bias were generally unaddressed in studies, and there was often inadequate information for generalising findings to other contexts. However, methodological approaches tended to be sound and reported results related to the stated research aims. A full overview of the quality of literature underpinning each theme is available in the Supplemental Materials (Table S2).

### 3.3 Overview of Findings

This scoping review identified that for people with intellectual disability, health literacy is not simply an individual cognitive skill but a relational and situated practice, shaped by interpersonal relationships, organisational practices and broader socio‐political contexts. The primary research question is answered in Tables 2 and 3 as we charted the experiences of people with intellectual disability. Furthermore, Tables 2 and 3 also address the secondary research question through charting the experiences of support networks and healthcare professionals (HCPs) and the social and environmental contexts shaping their interaction.

**Table 2:** Factors that influence how people with intellectual disability, their supporters and HCPs practice health literacy.

Dimension	Group	Theme	Examples from the literature
Accessing	People With Intellectual Disability	Exclusion from information	People with intellectual disability wanted opportunities to engage with health information [33, 34, 35], but were often excluded from health promotion initiatives [36, 37, 38, 39, 40, 41, 42, 43, 44, 45, 46, 47, 48, 49, 50, 51, 52, 53, 54]. Exclusion reflected structural and attitudinal barriers, where healthcare was treated as reactive rather than preventative. It was most evident in sexual and reproductive health, where stereotypes of people with intellectual disability as asexual or ‘childlike’ constrained their entitlement to information and opportunities for participation [39, 40, 41, 45]. When information was provided, it was typically risk‐focussed (e.g., STDs and pregnancy) rather than supporting broader understandings of relationships [40, 55]. Findings demonstrated that exclusion stems less from resource gaps than from societal assumptions and institutional practices that position people with intellectual disability outside normative health citizenship, reinforcing historical disempowerment and reactive, deficit‐framed health literacy [40, 56].
		Tailored and empowered approaches to information	Tailoring health information to individual needs improved access for people with intellectual disability. Accessible formats such as Easy Read, visual supports and digital tools enhanced engagement, though barriers including limited digital literacy, device access and communication partners constrained participation [56, 57, 58, 73, 104]. Some people with intellectual disability preferred to seek information independently, but their accessibility needs were often overlooked [58, 60]. For non‐speaking communicators [25], limited communication partners restricted ability to engage with healthcare systems [61]. Beyond accessibility, co‐design was critical for ensuring information was relevant and empowering, whereas its absence risked reproducing complexity and irrelevance [62, 63, 64].
	Supporters	Facilitating access to information	Supporters mediated health information by sourcing content [65, 66], encouraging engagement with HCPs [67] and adapting materials to enhance accessibility. Their influence lay on a spectrum between facilitation and gatekeeping, where protective or simplifying roles could restrict autonomy and direct engagement [25, 41, 60, 68], which at times positioned them as gatekeepers rather than facilitators [33, 47, 60, 69, 70]. Limited communication with families further created gaps in shared understanding, underscoring that health literacy is a relational rather than individual process [71].
		Resources and education	Supporters wanted training and guidance from health services to facilitate how they support people with intellectual disability to access information [33, 43, 44, 47, 65, 66, 69, 72, 73, 74, 75]. In the absence of such support, they relied on personal experience, creating uncertainties in the accuracy and consistency of information shared [76] [33, 77]. Accessible formats such as plain English, videos and demonstrations supported both individuals and supporters, particularly when supporters themselves faced informational barriers [25, 36, 63, 78]. Collaboration between supporters and information designers highlighted that effective resources need to be relational, not just individual, to ensure relevance and equitable access [41, 69].
	Healthcare Professionals	Source of information	General practitioners and pharmacists were key sources of health information for people with intellectual disability and their supporters [35, 79, 80, 81, 82]. However, HCPs sometimes acted as gatekeepers, limiting access despite their responsibility to provide accessible information [43, 52, 63]. Family supporters, in particular, faced unequal access to health resources compared with paid staff, underscoring structural inequities in how information circulates [69]. Health services that provide training and information to supporters, particularly family supporters, strengthened the capacity of supporters to provide more information [33, 69].
		Equipping mainstream professionals	Specialist HCPs regularly used accessible materials [83, 84], whereas mainstream professionals were often unaware of or failed to adopt tools such as Easy Read or AAC [61, 63, 71, 84, 85]. This gap reflected not only resource limitations but also systemic barriers such as time pressures and lack of training [43, 52] [71, 86]. Many HCPs lacked the skills to effectively promote health and share information with people with intellectual disability [6, 35, 43, 77, 79] [81], highlighting the need for structural investment in training and resources to achieve equitable practice.
Understanding	People With Intellectual Disability	Tailored delivery	Adapting communication to individual preferences, including for non‐speaking communicators, improved understanding [25, 38, 40, 58, 60, 70, 72, 87] and allowed for greater independence [85, 88]. Experiential learning also supported comprehension [33, 40, 42, 56, 58, 89, 90, 91, 92, 93, 94]. These practices positioned health literacy as relational and ongoing, embedded in daily interactions rather than a one‐off transfer of information [6, 84, 93, 95].
		Psychosocial processes	Building self‐efficacy, confidence and a sense of safety was important [80, 89] and often achieved through rapport [96]. When information was not understood, it caused anxiety [59], and past negative experiences made people hesitant to admit confusion [41] or try new activities [89]. These dynamics highlight how health literacy is shaped not only by skills and resources but also by emotional and relational contexts.
	Supporters	Literacy mediation	Supporters played a central role in preparing people with intellectual disability for healthcare encounters and shaping health understanding through role modelling—sometimes enabling, but at times limiting autonomy [63, 65, 67, 78, 85, 87, 92, 97, 98, 99]. They also facilitated tailored communication with HCPs [97], though dominance in interactions could restrict direct engagement [89]. Despite this centrality, most resources were not designed to reflect the collaborative and relational nature of health literacy, overlooking how understanding emerges between people, supporters and the materials that mediate communication [23, 78, 92].
		Supporters are not medical experts	Supporters reported discomfort with expectations to support people to understand information from—or to question—HCPs [47, 65, 87]. They relied on HCPs to support their understanding of health management plans [69] and wanted to be equipped to continue discussions outside of health services [41, 50, 54, 81, 92, 98, 100]. This highlights a tension as supporters were positioned as essential literacy mediators but lack the medical expertise or structured support to fulfil this role effectively.
	Healthcare Professionals	Capacity to facilitate triadic communication	Healthcare professionals required stronger skills in recognising diverse communication needs and adapting strategies accordingly [12, 67, 71, 86] [88]. Tools such as Easy Read, hospital passports and teach‐back supported shared understanding [57, 62, 81, 88, 97, 101]. Crucially, professionals were responsible for ensuring that information was accessible to both people with intellectual disability and their supporters [35, 70, 101], highlighting the inherently triadic nature of health communication where meaning is co‐constructed across patients, supporters and clinicians.
Appraising	People With Intellectual Disability	Trust, rapport and autonomy in information exchange	Trust in the person delivering health information was central to whether people with intellectual disability viewed it as reliable [56, 59, 67, 71, 89, 97, 102]. Mainstream services often reproduced historical disempowerment by limiting opportunities to question or decline care [103]. Easy Read resources, while intended to increase accessibility, were critiqued for rarely depicting genuine choice or autonomy and instead presenting compliance with health advice as the only option [40, 62, 85]. When people with intellectual disability were involved in co‐design, information was not only more accessible but also affirmed their autonomy and capacity to critically engage [38, 40, 42, 49, 50, 92, 94, 104].
	Supporters	Upholding the right to disagree	Supporters play a key role in enabling people with intellectual disability to question health advice and assert their rights, positioning disagreement as a legitimate and necessary dimension of health literacy [50, 103].
	Healthcare Professionals	Rapport‐building skills	Continuity of care and the cultivation of trust with people with intellectual disability and their supporters enhanced the credibility and acceptance of health information [59, 97], underscoring rapport as a foundational skill for effective health literacy practice.
Using	People With Intellectual Disability	Confidence and motivation	Acting on health information depended not only on access but on motivation and confidence [50, 54, 56, 66, 92, 94]. Supporters were important motivators [97], while accessible, relevant information helped people with intellectual disability feel comfortable, overcome fears and build the confidence needed to apply knowledge in practice [6, 12, 23, 35, 42, 79, 95].
		Independent and supported decision‐making	Health literacy extended beyond receiving information to actively exercising choice and autonomy. Supporting self‐efficacy and self‐advocacy helped counter historical disempowerment [58, 66, 82, 92, 103, 105]. Direct involvement in decisions [89], access to tailored resources [58, 59, 63, 64, 66, 80] and opportunities to apply knowledge in everyday contexts such as cooking or managing health behaviours upheld autonomy [39, 50, 104, 106, 107, 108]. Yet a persistent gap remained between understanding information and being supported to act on it [39, 90, 109, 110].
		Relevant information	Health information was more likely to lead to action when it was personally meaningful and connected to individual goals or circumstances [25, 36, 43, 56, 64, 78, 82, 97, 102]. Linking information to hobbies, interests, employment or other valued life domains encouraged uptake of behaviours and future planning [42, 56].
	Supporters	Ethical dilemmas	Supporters faced tensions between respecting autonomy and fulfilling their duty of care [38, 48, 65, 68, 89, 110, 111]. While promoting health in line with individual preferences was valued [38, 69, 110], difficulties arose when people with intellectual disability chose behaviours perceived as unhealthy [69].
		Gatekeeping	Supporters often shaped and constrained the options available to people with intellectual disability [52, 69, 76, 111, 112]. When supporters lacked health knowledge or confidence, this limited the quality of choices offered (e.g., restricted access to healthy food) [38, 46, 52, 60, 69, 74, 76, 111]. Their own anxieties and health beliefs further influenced decision‐making, sometimes reinforcing dependency rather than autonomy.
		Confidence in role	Supporters' motivation and confidence were critical to enabling autonomy in decision‐making [89]. Tools and resources that provided guidance, structured ways to track health information, and strategies to promote autonomy helped strengthen supporters' capacity and, in turn, enhanced the motivation of people with intellectual disability [38, 54, 57, 67].
	Healthcare Professionals	Supporting choice	Strong relationships with HCPs fostered shared decision‐making [80], yet implicit biases often led HCPs to bypass individuals and defer to supporters, undermining autonomy [57, 79, 101] [63, 97]. While some people with intellectual disability preferred clinicians to make decisions, they still valued being informed. Training and accessible resources are therefore essential to equip HCPs to facilitate inclusive, informed decision‐making [61] [60, 97].

**Table 3:** Social and environmental, situational and personal determinants of health literacy.

Dimension	Theme	Description
Social and Environmental	Upholding the right to information and autonomy	Protecting the rights of people with intellectual disability to make their own health choices was central to health literacy [39, 47, 51, 68, 89, 113, 116, 129]. International frameworks such as the UNCRPD and national legislation like Ireland's Disability Act (2005) represent formal efforts to uphold these rights in response to historic exclusion [52, 59, 63, 81, 84, 102, 113, 116, 130]. Yet entrenched societal attitudes, shaped by histories of disempowerment, continue to position people with intellectual disability as incapable or undeserving of information, leading health and disability systems to assume they neither need nor would use it [40, 51, 61, 72, 87, 96, 116].
	Media as a source of information	Media, particularly the internet, social media and television, shaped how people with intellectual disability and their supporters accessed health information, especially in areas like sexual health [12, 40, 67, 79, 129]. While these platforms expanded access, they also influenced supporters' interpretations of health information and advice [111, 112]. However, the complexity of online environments created risks of misinformation and disinformation, underscoring the need for support in evaluating digital health information [39, 116, 129].
	Schools as a resource for health literacy	Schools played a central role in shaping health literacy, not only for students with intellectual disability but also within their families [39, 55, 78, 113, 129]. Teachers acted as key facilitators of information access, though at times also as gatekeepers who constrained opportunities for engagement [44, 55, 107, 116].
Situational	Social and familial networks	Social networks provided opportunities to apply health information and sustain motivation [6, 69, 90], yet people with intellectual disability often had fewer opportunities for social connection than their peers without disability [33, 90, 129]. Families were also central to health literacy, requiring resources to meet both the health needs of those they support and their own well‐being [113].
	Disability sector policies	Disability sector policies that prioritised health promotion enabled resources for support staff and those they support, fostering opportunities for informed health choices [34, 39, 51, 58, 65]. Yet risk‐averse cultures, underfunded services and unclear responsibilities across multiple providers often constrained everyday decision‐making and created gaps in health information [40, 76, 84, 95, 111, 131].
	Health sector policies	Health sector policies mandating that providers meet the communication needs of people with intellectual disability supported more inclusive care [62, 94]. HCPs benefited from employer‐supported training and health systems that accommodated both individuals and their supporters [60, 61, 62, 88, 97]. System‐level improvements, such as protected time and adequate resources, further enabled equitable health literacy practices.
Personal	Intersectional identities	Intersecting identities shaped how people with intellectual disability accessed and used health information. Culturally and linguistically diverse individuals faced compounded stigma and barriers [33, 42], while gender and sexuality further influenced experiences, with LGBTQIA+ people often navigating information within heteronormative contexts [40, 47].
	Intellectual disability is heterogeneous	Accessible and enabling environments require recognition of the heterogeneity of intellectual disability. Experiences of disability and health varied both between individuals and across the lifespan, underscoring the need for flexible, tailored approaches to health literacy [56, 60, 69, 70, 76, 77, 89, 91, 92, 95, 116, 129].

As illustrated in Table 2, there was a range of factors that influence how people with intellectual disability, their supporters and HCPs practice health literacy. Across the phases of accessing, understanding, appraising and applying information, all three groups had active roles. People with intellectual disability were central to decision‐making but often constrained by exclusionary structures [36, 37, 38]; supporters acted as both facilitators and gatekeepers [69, 72]; and HCPs were trusted sources whose effectiveness depended on communication skills and systemic support [69, 71, 72, 86]. These findings highlight health literacy as a relational practice that relies on collective action rather than individual skill.

The capacity to engage with information was shaped by how access was facilitated. Supporters and HCPs acted as central mediators of information, with supporters adapting materials, encouraging engagement or gatekeeping access depending on their own confidence, training and resources [65, 78]. HCPs, though consistently trusted sources, often lacked the interpersonal and triadic communication skills needed to make information accessible [62, 101]. These dynamics demonstrate that literacy mediation is rarely a one‐way process but rather a shared negotiation of meaning between people with intellectual disability, their supporters and HCPs.

Beyond accessibility, psychosocial processes such as trust, rapport, confidence and autonomy determined whether information was perceived as reliable and acted upon [67, 97, 102]. While autonomy was frequently constrained by systemic assumptions of incapacity, enabling relationships and rights‐based approaches created space for informed choice and the right to disagree. At the same time, the influence of power and gatekeeping was evident: HCPs often retained authority in decision‐making, while supporters' anxieties and knowledge gaps structured the range of choices available to people with intellectual disability [57, 79].

These findings also highlight the importance of broader contexts. Inclusive schools, services and community environments facilitated opportunities to practise health literacy [39, 113]. Legislation and sector policies determined whether such opportunities were sustained or undermined by risk‐averse or under‐resourced systems [40, 111]. By contrast, personal determinants were less consistently examined; some studies addressed intersectional experiences relating to gender, sexuality [40] or cultural diversity, but gaps remained for culturally and linguistically diverse groups, socio‐economic status and other known influences on health literacy [72].

Taken together, the themes illustrate that health literacy for people with intellectual disability is best understood as an interactive and socially distributed process. They extend the integrated model by showing that exclusion, tailored approaches, psychosocial processes and gatekeeping are not discrete issues but interdependent dynamics, emphasising that health literacy is co‐produced through the interplay of individuals, relationships and contexts.

To note, the quality of evidence for most themes was moderate. Although methodological limitations exist, the consistency of themes across diverse contexts suggests conceptual validity. Furthermore, the theme of resources and education for support networks facilitating understanding was low, highlighting that more work is needed to understand training and education needs and that this theme should be interpreted cautiously. Finally, themes of trust, rapport and autonomy in information exchange for people with intellectual disability, rapport‐building skills for HCPs, and the role of health sector policies were all developed on high‐quality literature. As such, we have confidence that these themes are supported by a robust evidence base.

## 4 Discussion

This scoping review identified barriers and facilitators to health literacy for people with intellectual disability, their supporters and HCPs. Findings reinforced the perspective that health literacy is a shared and distributed resource *and* responsibility. People with intellectual disability find, access, appraise and apply health information in collaboration with others, making tailored, accessible and multimodal information important. However, it also requires attention to how information is used and shared in collaboration, the formation of trusting and reliable relationships and the systems in place to support health literacy practices [56, 68, 89, 114, 115]. The way information was used and shared between people with intellectual disability, their supporters and their HCPs was related to organisational policies that impacted the right to information and choice [47]. Such policies often reflected ongoing stigmatising and discriminatory assumptions that do not position people with intellectual disability as information users or as a group that can or should be empowered and supported to make their own informed decisions [116].

### 4.1 Refinement of Sørensen et al.'s Integrated Model of Health Literacy

**Figure 3:**
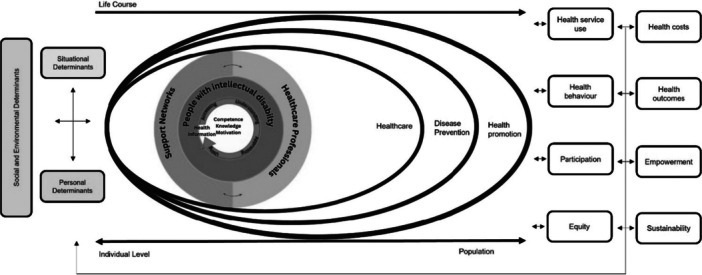
Visualisation of distributed health literacy between people with intellectual disability, their support networks and healthcare professionals.

Sørensen's integrated model provided a useful foundation for charting health literacy processes for people with intellectual disability. However, the evidence highlighted the value of making its relational dimensions more explicit. Studies consistently showed that individuals were not working alone at the centre to access, understand, appraise and apply information. Instead, these processes were dynamic social interactions between people with intellectual disability, their supporters and HCPs [6]. On this basis, the evidence indicates that models of health literacy may be strengthened by explicitly acknowledging relational and distributed processes, rather than conceptualising supporters and HCPs as external ‘situational’ determinants within Sørensen et al.'s framework. We visualise this process in Figure 3, demonstrating that the health literacy of people with intellectual disability is influenced by the interactions of the supporters and HCPs around them. This relational perspective remains consistent with Sørensen et al.'s model but adds clarity by emphasising that each group's engagement shapes health literacy processes and is influenced by both shared and individual environmental resources.

This relational aspect is further underpinned by findings that trust and rapport shaped whether people with intellectual disability viewed information as reliable. Information was more trusted when it came from HCPs or supporters with established positive relationships [59, 71]. Rapport was fostered through active listening, validation, plain language, accessible communication and shared decision‐making [59, 97]. Time was critical for meaningful interactions but constrained by systemic barriers such as staffing and remuneration. High levels of trust in supporters and HCPs significantly influenced how people with intellectual disability engaged with and acted on health information [43, 52].

Evidence relating to support networks had two key aspects: the health literacy of support networks themselves and the resources they need to support people with intellectual disability with their health literacy. Supporters sometimes restricted the choices available to people with intellectual disability, often due to their own attitudes about what information was appropriate, gaps in accessible resources, poor communication or limitations in their own health literacy [47, 63]. As such, improving health literacy capacity among supporters is needed. There were some examples of healthcare services aiming to address these issues by supporting not only the person with intellectual disability but also their supporters. For example, some interventions included supporters as joint participants in healthy eating classes and exercise activities and ensured their participation and understanding in medication administration instructions [81, 88]. HCPs were also identified as a common source of information for both people with intellectual disability and their supporters, with evidence highlighting the value of resources and strategies that facilitate accessible and triadic communication.

Furthermore, situational and societal circumstances shaped the health literacy process and outcomes for people with intellectual disability. This was most notable in the availability and accessibility of sexual and reproductive health information, which was impacted by societal misconceptions that people with intellectual disability are uninterested in intimate relationships or that this information is inappropriate to provide [47]. LGBTQIA+ people with intellectual disability are further affected by heteronormative societies, often resulting in the denial of their identities [40, 47]. Assumptions result in policies and practices within healthcare, disability support and family settings that overlook individual‐specific informational needs of people with intellectual disability.

### 4.2 Implications of Findings for Policy and Practice

The findings echo recommendations from the Australian Disability Royal Commission (DRC), including the development of accessible information and communications to reduce disparities in information access, informed consent and decision‐making participation. Public health and disability support sectors can invest in co‐creating and co‐developing multimodal accessible resources on a range of health topics [63]. This includes written easy‐read information, visual demonstrations of procedures or disease progression and workshops for public health messaging. This work involves nuancing the health needs of the general population with the priorities of people with intellectual disability and their supporters. Eliciting these priorities can be achieved through engaging with the community to understand their values and lived experiences, building trust from the community towards the healthcare sector and finding opportunities to integrate health needs with community values. Indeed, co‐designing information with both people with intellectual disability and supporters facilitates effective user strategies for finding and engaging with information [62, 63]. Furthermore, it can address the tendency for health organisations to overlook their informational responsibilities to supporters of people with intellectual disability [117].

Tailored information alone is not sufficient. Our review suggests that practitioners can better support health literacy for people with intellectual disability through their interpersonal skills and through challenging any consciously or unconsciously held stigmatising attitudes. Attitudes have a direct role in the opportunities made available to people with intellectual disability to use information to make choices. Reflective practice for HCPs and supporters is a person‐level mechanism for changing current practices and challenging biases that limit autonomy for others [118]. At the organisational level, healthcare services and disability support organisations have a responsibility to uphold information rights by ensuring there are policies that facilitate access to health information for people with intellectual disability and initiatives to include people in their daily health choices [24, 65].

Much of health literacy develops outside health services, so government initiatives in social spaces (e.g., gyms, schools, community centres and online groups) can improve access to information [118]. This review and wider public health literature highlight schools as key sites for building foundational health literacy [119]. Schools help foster awareness of misinformation and skills in critically assessing health information, creating early opportunities for decision‐making by people with intellectual disability [107, 120, 121]. Leveraging these spaces may involve integrating health literacy into curricula with tailored content, accessible tools and easy‐read or visual campaigns. Partnerships between health services and schools or community organisations can support co‐design initiatives that benefit young people with intellectual disability. In Australia, government‐funded foundational supports provide community‐based disability supports. Embedding health literacy in such initiatives can strengthen community understanding of disability and disability health, enhancing overall public health literacy.

Likewise, supporters have a significant role to play in the daily health choices people with intellectual disability make (e.g., exercising, making lifestyle changes and taking medication) [48, 120, 122, 123, 124]. At present, it seems they are often overlooked in their roles, with few initiatives for assisting and sustaining them as they support others with their health. Government and disability sector initiatives can focus on providing the right education and training opportunities to improve health literacy among supporters. Indeed, in Australia, formal education and training for support workers in disability services is not universally mandated, and training that may be undertaken focusses on basic communication or person‐centred care. Health and health literacy are not often considered a central component of support worker mandates, despite being a common function of support worker roles. Recognition of daily health support as part of their roles and mapping the health literacy needs of supporters is first needed to facilitate the creation of meaningful training opportunities. From here, disability services can ensure their employees are supported on an ongoing basis to navigate health services, understand basic preventive health, and support the dignity of risk with the duty of care in their roles.

Given that the reviewed literature conceptualises health literacy as a shared practice, some studies argued that literacy measures are more informative when they move beyond functional and individual assessments to include social resources, capacity to use these resources, autonomy, and alignment with personal goals and values. Recent approaches have developed system‐oriented strategies that focus on organisational responsibilities to develop, maintain and promote individual and population health literacy [125]. The Institute of Medicine of the National Academics proposed a list of attributes that health literate organisations should adopt to benefit everyone navigating the healthcare system [126]. These include responding to the individual needs of diverse populations, avoiding stigmatisation of populations, promoting successful interpersonal communication and ensuring equitable access to health information.

Healthcare organisations can implement processes to assess their current health literacy, engage with stakeholders such as people with intellectual disability and support organisations, and develop plans to improve their health literacy outcomes. HCPs can benefit from clear guidelines for meeting each of these attributes, with explicit provision of strategies for working with patients with intellectual disability [126]. Moreover, transitioning from child to adult services comes with many challenges for people with intellectual disability, including loss of information and support. Creating measures for monitoring a person's health literacy readiness and needs related to transitioning services may improve experiences [127]. Critical for the successful implementation of organisational health literacy interventions is the presence of advocates for change, support from leadership and a supportive management structure and culture for innovation [126].

### 4.3 Limitations and Future Directions

This scoping review contributes to a comprehensive understanding of the social context of health literacy for people with intellectual disability. However, it is not without limitations. Health literacy is a broad concept that has been developed from fields of health promotion and health education. We used inclusion criteria and search terms that reflect this diversity. While the screening process aimed to ensure any relevant texts were included, some relevant texts may have been missed in the search. We also excluded articles which were not available in English, which resulted in a majority of included studies coming from high‐income and English‐speaking countries.

Almost a third of included studies were rated poor quality, often due to limited reporting on participants or a lack of reflexivity. This weakens the strength of inferences, especially for themes based on lower‐quality evidence, such as supporter training and education, which should be interpreted cautiously, as they may reflect context‐specific practices or bias. In contrast, themes supported by higher‐quality studies, trust, rapport and autonomy in information exchange, are more robust. The predominance of moderate‐ and low‐quality studies underscores the need for more rigorous designs, clearer reporting and stronger attention to ethics and sampling. We identified similar issues in a prior scoping review [128], reiterating that efforts are needed to ensure future work reports on these issues more explicitly.

Further work is needed to develop measurement and assessment tools for health and disability organisations to assess their current health literacy practices and support practice change in these sectors. Most existing measurements focus on healthcare services rather than providing frameworks for disability support organisations. They may not be fit for purpose, as they do not consider processes such as support and historic disempowerment, which have important implications for health literacy with people with intellectual disability. They also do not provide an avenue for patients to assess how health literate they believe an organisation to be, instead focussing on organisational self‐assessment.

Evidence for personal determinants influencing how people with intellectual disability, their supporters and HCPs work together to find and use information was lacking. Outside of exploring experiences of LGBTQIA+ groups, there was limited literature on culturally and linguistically diverse communities and no literature regarding the health literacy experiences of those in rural communities or the role socio‐economic status might play in access to health literacy opportunities. Intersectional experiences are impacted by overlapping discrimination. Understanding these nuanced viewpoints informs how services can support health literacy for all people with intellectual disability. Further research may utilise participatory approaches that partner with multicultural or First Nations organisations or focus on capturing the experiences of people with intellectual disability from diverse backgrounds. Comparative study designs can further enhance our understanding of the barriers and facilitators to practising health literacy across different settings, including rural and metropolitan, or lower and higher socio‐economic statuses. From here, policy audits can be used to understand if the current policy adequately meets the intersectional needs of people with intellectual disability.

## 5 Conclusion

Health literacy for people with intellectual disability is a relational process co‐constructed between individuals, supporters and HCPs, shaped by the social, organisational and policy contexts that determine access and use of information. This review extends Sørensen et al.'s model by foregrounding health literacy as distributed rather than individual, highlighting the roles of trust, autonomy and systemic facilitators. The findings show that supporters often act as literacy mediators, while HCPs remain central sources of information, yet both groups require resources, training and structural support to fulfil these roles without reinforcing bias or disempowerment. The novel contribution of this review lies in establishing health literacy for people with intellectual disability as a rights‐based, shared responsibility, with implications for developing health‐literate organisations, advancing supporter education, embedding inclusive communication in healthcare systems and strengthening the evidence base through more rigorous research.

## Author Contributions

**Maryann Barrington:** conceptualisation, methodology, writing – original draft, interpretation, writing – review and editing, project administration, data curation, validation. **Karen R. Fisher:** funding acquisition, writing – review and editing. **Ben Harris‐Roxas:** funding acquisition, interpretation, writing – review and editing. **Julian N. Trollor:** funding acquisition, interpretation, writing – review and editing. **Catherine Spooner:** interpretation, writing – review and editing, funding acquisition. **Janelle Weise:** conceptualisation, interpretation, writing – review and editing. **Julie M. Christensen:** data curation, interpretation, writing – review and editing.

## Conflicts of Interest

The authors declare no conflicts of interest.

## Supporting information

Supplemental Materials_Resubmission.

## Data Availability

Data sharing is not applicable to this article as no datasets were generated or analysed during the current study.
