# Supplementary material for: Distributed Health Literacy Among People With Intellectual Disability, Their Supporters and Healthcare Professionals: A Scoping Review
Source: Health Expect. 2026 Mar 18;29(2):e70548. doi: 10.1111/hex.70548 (PMC13080893; doi:10.1111/hex.70548)
Supplement: Supplementary file 1 — Supplemental Materials_Resubmission. [file HEX-29-e70548-s001.docx]

**Figure S1**

*Example search strategy: EMBASE*

| **Population** | (“intellectual disab*”.ti,ab OR “learning disab*”.ti,ab) |
| --- | --- |
|  | ***AND*** |
| **Concept** | (“health literacy”.ti,ab OR “distributd health literacy”.ti,ab OR “health capabilit*”.ti,ab OR “health promotion”.ti,ab OR “health education”.ti,ab OR “health literacy responsiveness”.ti,ab OR “organisational health literacy”.ti,ab OR “organizational health literacy”.ti,ab OR “patient-centered communication”.ti,ab OR “patient-centred communication”.ti,ab OR “health information”.ti,ab OR “patient literacy”.ti,ab OR “patient education”.ti,ab OR “critical health literacy”.ti,ab OR “health communication”.ti,ab OR “information seeking”.ti,ab OR “health knowledge”.ti,ab) |

**Note.** No search strategy was used for context as all contexts were considered.

**Table S1**

*Characteristics of included sources.*

| **Authors (Year)** | **Country** | **Source Type** | **Aims** | **Method** | **Population(s)** |
| --- | --- | --- | --- | --- | --- |
| Anderson & Wickham (2022) | Ireland | Journal Article | The aim was to determine how social care managers working within an intellectual disability care setting in Ireland perceived health promotion and their role within it. | Qualitative. Semi-structured interviews and thematic analysis. | Support staff |
| Asher et al. (2024) | Australia | Journal Article | The present study aimed to describe the process used to plan, develop and refine the program, which was named the Food and Lifestyle Information Program (FLIP), and pilot study. A secondary aim was to describe the outcomes of the planning and development phases. | Qualitative. Codesign/Community engaged design, including consultations. | Support staff People with intellectual disability |
| Ashworth & Carton (2022) | United Kingdom | Journal Article | This paper aims to provide an overview of the development, pilot and evaluation of a programme aimed at increasing awareness of the intricacies and risks of sexual health, intercourse and relationships. | Quantitative Pre-post comparison within-group design measuring changes in sexual attitudes and knowledge | People with intellectual disability |
| Bains & Turnbull (2020) | United Kingdom | Journal Article | The current study aimed to gather perspectives of people with intellectual disability and their carers on how uptake of healthy eating and physical activity could be improved amongst adults with intellectual disability. | Qualitative Semi-structured interviews based on social cognitive theory | People with intellectual disability Support staff and family carers |
| Bergström et al. (2014) | Sweden | Journal Article | The aim of this study was to explore barriers and facilitators in the implementation of a health course for adults with mild or moderate intellectual disability. | Qualitative Analysis of course sessions, group discussion with course leaders and evaluation notes from the course leaders | People with intellectual disability |
| Bergström et al. (2011) | Sweden | Journal Article | The aim of this article was to explore variation in views among managers and caregivers on the role of staff in health promotion. | Qualitative - phenomenographic approach and qualitative content analysis. Semi-structured interviews. | Support workers and managers |
| Bollard (2017) | United Kingdom | Journal Article | The study aimed to explore how men with mild-to-moderate intellectual disability understood and perceived their health and what health promotion messages they acted upon. | Qualitative Participatory approach, participants formed a steering group using one-to-one interviews. Semi-structured interviews. | People with intellectual disability |
| Borthwick et al. (2021) | Scotland | Journal Article | Through conducting semi-structured interviews, the present study sought to understand the facilitators and barriers that caregivers faced when implementing health promotion advice. | Qualitative Semi-structured interviews | Family caregivers Support workers |
| Broberg & Temple (2014) | Sweden | Book Chapter | - | - | - |
| Cardell (2015) | United States of America | Journal Article | The purpose of this article is to acknowledge an alternate, or at least supplemental, direction for promoting the health of people with ID. By analyzing the data through the lens of  occupational science, I suggest that the results support the  proposal that inequities experienced by this group go beyond  individual behaviors and health care delivery and are related  to aspects of social justice. | Qualitative - information was gathered through direct elicitation of adults with ID and through observation. | People with intellectual disability |
| Chinn (2019) | United Kingdom | Journal Article | The current study addresses a gap in the literature to explore how health consultations involving patients with intellectual disabilities can be viewed as containing lit‐ eracy events in which different practices involved in interaction with texts are made evident. | Qualitative - conversation analysis of video recordings of patients with intellectual disability attending a health check with primary care clinicians. | People with intellectual disability Primary care clinicians |
| Chinn (2017) | United Kingdom | Journal Article | This article examines Easy Read health information designed for people with intellectual disabilities as a cultural artefact that can tell us about ‘the cultural, discursive and relational undergirdings of the disability experience | Qualitative - textual analysis of easy read pamphlets produced by health departments | People with intellectual disabiltiy |
| Chinn (2019) | United Kingdom | Journal Article | Therefore, the aims of this study were to understand from those immersed in the design and creation of Easy Read health information (ERHI) (i) which practices they prioritised in the creation of ERHI; (ii) what elements they felt were most important in deciding on the content of ERHI; (iii) how they anticipated how their materials might be used in real-life situations. | Qualittive study - semi-structured interviews. | Producers of Easy Read information |
| Chinn & Rudall (2021) | United Kingdom | Journal Article | The aim of this study was to establish, within naturally occurring health consultations, who HCPs select to answer their questions during health checks – patients with ID or their companions – and who actually answers. | Qualitative - conversation analysis of video recordings of patients with intellectual disability attending a health check with primary care clinicians. | People wiht intellectual disability General practitioners Support networks |
| Chou et al. (2020) | Taiwan | Journal Article | This paper primarily evaluates the outcomes of a two-year intervention to promote sexual and reproductive health knowledge/positive attitudes and quality of life for adults with ID. | Mixed methods Qualitative - interviews and focus groups Quantitative - two year evaluation investigating attutudes toward sexual health, sexual knowlede, and QoL of adults with ID. | People with intellectual disability Parents Support Workers |
| Colarossi et al. (2023) | United States of America | Journal Article | To better understand professionals’ current communication practices, attitudes, and needs related to sexual and reproductive health (SRH) education for adolescents and young adults with ID, we conducted formative research for a broader initiative, Project SHINE (Sexual Health Innovation Network for Equitable Education – with Youth with Intellectual Disabilities). | Mixed methods Project shine is a 3-year initiative to create and test innovative sex education tools for young adults aged 16-24 years old. Qualitative - Staff conducted exploratory research with focus groups discussions with youth with ID and their parents. Quantitative - survey measuring attitudes | Service Providers (licensed and unlicensed) |
| Colarossi et al. (2023) | United States of America | Journal Article | We conducted exploratory research to gather input from a diverse group of youth with disabilities and their guardians about needed learning modalities and sexual health content areas. | Qualitative Focus Groups  Project shine is a 3-year initiative to create and test innovative sex education tools for young adults aged 16-24 years old. | Youth with intellectual disabilities Parents of youth with intellectual disabilities |
| Dam et al. (2023) | Austria | Journal Article | The “LUCHS-Gesundheitsinformationen (Health Information)” project aimed to improve knowledge transfer of health information for people ID by creating a website prototype. | Qualitative Unstructured interviews on two ocus groups | People with intellectual disability Their caretakers |
| Davies & Duff (2001) | United Kingdom | Journal Article | The aim of the present study was to review the uptake and knowledge of women with ID living in the community of breast screening programmes. | Quantitative Survey | Community care workers and people with intellectual disability |
| Davis et al. (2016) | Australia | Journal Article | To explore the level of understanding of asthma medication use of people with ID who self-administer their inhaled medications, in order to inform future educational support. | Qualitative Interviews - semi-structured | People with intellectual disability |
| Dins & Keeley (2022) | Germany | Journal Article | People with profound intellectual and multiple disabilities (PIMD) are often overlooked in health literacy research. This article aims to address this by presenting the approach and main findings of the research project 'Communicating in the Crisis' or ComCi. This project focuses on the vulnerabilities and the health literacy needs of people with PIMD and their supporters. | Multimethod qualitative approach Review of the current state of research Delphi study Online Survey Ethnographic case studies | Academics Support service professionals |
| Doyle (2008) | United Kingdom | Journal Article | Provide information about how to improve sexual health education for young people with disabilities. | Commentary/literature summary | None |
| Elinder et al. (2018) | Sweden | Journal Article | This study evaluated effectiveness of a structural health intervention, a study circle for paid carers aiming to improve health promotion work routines for residents, and explored barriers and facilitators in he implementation process. | Quasi-experimental Questionnaire Inductive qualitative method | Caregivers |
| Feldman et al. (2016) | Canada | Journal Article | The objective of this study was to determine whether adults with intellectual disabilities could learn basic biological and health concepts to gain a better understanding of their body, illnesses and good health practices. | Quantitative Randomized control design | People with intellectual disability |
| Finlay et al. (2015) | United Kingdom | Journal Article | The aim of this study was to carry out a preliminary investigation of how SHE is delivered in practice to young people with intellectual disabilities. We were particularly interested in the barriers that make delivery difficult, the perceived need for such provision, the institutional and social context, and the challenges faced by teachers and community workers. | Qualitative 4 videos of sexual health educations recorded and conversational analysis applied | People with intellectual disability |
| Fjellstrom et al. (2023) | Sweden | Journal Article | This study aimed to explore the support staff's experiences regarding the feasibility of adapted web-based exercise for people with intellectual disabilities. | Qualitative Semi-structued interviews | Support networks |
| Flood & Henman (2015) | Ireland | Journal Article | The aim of the project was to gain insight into the knowledge and views that people with intellectual disabilities had of the medication use process in their vulnerable population. | Qualitative Case Study | People with intellectual disability |
| Geukes et al. (2019) | Germany | Journal Article |  | Theoretical discussion | People with intellectual disability |
| Gilbert et al. (2007) | United Kingdom | Journal Article | Living with cancer pack was created to support the understanding of people with intellectual disability about cancer and cancer treatment and prevention. This paper reports on the findings of an evaluation study commissioned as part of the original grant from the Big Lottery Fund. | Mixed methods Focus groups Questionnaire Telephone interviews | People with intellectual disability Practitioners Care managers |
| Grove et al. (2018) | United States of America | Journal Article | This study demonstrates the use of Universal Design for Learning (UDL), an educational framework, guidelines, and checklist tools to increase accessibility in HIPTeens, an evidence-based sexual risk reduction intervention. | Qualitative Assessing UDL design features in an evidence-based SH program for youth | People with intellectual disability |
| Hall et al. (2011) | United Kingdom | Journal Article | Our study adds to existing knowledge because it reports on the acceptability, usability, and potential utility of virtual reality as a means of providing health care-related information to people with intellectual disabilities . | Qualitative Participatory research Video recorded participants using VR Assessed retention for information | People with intellectual disability |
| Hanna et al. (2011) | Northern Ireland | Journal Article | The aim of this study was to examine how staff engaged in cancer prevention and health promotion activities and behaviours on behalf of people with ID. | Quantitative Exploratory descriptive study Questionnaire | Care facility staff |
| Hole et al. (2022) | Canada | Journal Article | This article centers the voice of self-advocates with ID regarding barriers to sexual health knowledge, sexual expression, and sexuality. | Qualitative description Interviews & content analysis | People with intellectual disability |
| Holly & Sharp (2014) | United Kingdom | Journal Article | This project sought to evaluate the logistic utility and acceptability of a CHD training programme. Broadly, the project intended to improve the health outcomes, health literacy and self-management of individuals with a learning disability. | Mixed method  Quantitative 10-item MCQ to assess knowledge Training Acceptability Rating Scale  Qualitative Informal feedback | Support staff |
| Jeyachandran et al. (2022) | India | Journal Article | The current study examines the perspective of sexual health education and the challenges faced by special educators in imparting the same to adolescents with intellectual disabilities. Exploring the factors that hinder effective sexual education, health, and safety of adolescents with intellectual disabilities is essential. | Qualitative description Semi-structured interviews | Special educators |
| Jobling (2001) | Australia | Literature Review |  | Literature review | People with intellectual disability |
| Joyce (2024) | Australia | Journal Article | This paper highlights gaps in a settings-and-systems-based approach to promoting the health and wellbeing of people with an intellectual disability, particularly with respect to workplace health promotion. | Literature review | People with intellectual disability |
| Kok & Akyuz (2015) | Turkey | Journal Article | This study aims to determine the educational needs of parents regarding the sexual development of their adolescent children with intellectual disabilities and to evaluate the effectiveness of relevant health education with the participation of the parents. | Mixed methods Interventional study Semi-structured interviews to determine content Self-efficacy, assessment of knowledge before and after, satisfaction were all measured | Parents of children with intellectual disability |
| Kuijken et al. (2016) | Netherlands | Journal Article | The aim of this qualitative study was to gain insight into the perspectives of people with mild to moderate ID on healthy living. | Qualitative study Semi-structured focus groups | People with intellectual disability |
| Kuijken et al. (2018) | Netherlands | Journal Article | This two-phase qualitative study aims to obtain an overview of stakeholders in the network of people with intellectual disabilities and their perceived facilitating and hindering factors, expectations, and perceived roles and responsibilities with regard to health promotion. | Qualitative 1) Stakeholder workshops to identify relevant stakeholders 2) Interviews to explore stakeholder views | Support networks |
| Kurt et al. (2024) | Turkey | Journal Article | This study aimed to identify the barriers experienced by nurses regarding communication for sexual health education for children with intellectual disbailities. | Qualitative In-depth interviews and thematic analysis | Nurses |
| Kurt & Kurtuncu (2024) | Turkey | Journal Article | The purpose of this study is to determine the effectiveness of an educational program implemented to support the sexual health and development of children with intellectual disabilities as well as on their mother's awareness of their children's sexual development. | Quantitative RCT Education versus control group Measured sexua development characteristics of children with ID (for the mothers) and sexual development knowledge assessment (for the children) | Children with intellectual disabilities Their mothers |
| Latteck & Bruland (2020) | Germany | Journal Article | To increase knowledge on HL concept for PwID we bring together two different research projects and describe the best practical example. Our primary aim is to answer the question regarding to what extent HL concepts are applicable for people with IDs or whether a target-group adaption is necessary. | Literature review | People with intellectual disability |
| Mauro et al. (2021) | Germany | Journal Article | The aim of the research project was to develop an intervention for people with intellectual disability that strengthens self-management competencies for initiating and maintaining physically active behaviour in everyday life. | Qualitative Participatory design of a physical activity intervention Interviews with PwID about the intervention when designing it and interviewed PWid and exercise 'buddies' after to evaluate it | People with intellectual disability Support networks |
| Lee et al. (2019) | Hong Kong | Journal Article | The current study aimed to explore family carers' experiences after participating in a 12-month school-based weight management via mHealth tools with interactive intervention. | Qualitative Focus group about participating in the weight management programme | Family caregivers |
| Lennox et al. (2004) | Australia | Journal Article | Described here is the development of an educatonal package designed to enhance communication and advocacy between the patient and their GP, or between the patient, their GP and the advocate. | Qualitative Reporting on the design and reception of a patient advocacy educational package. | People with intellectual disability Support networks |
| Llewellyn et al. (2003) | Australia | Journal Article | The objective of this study was to evaluate the efficacy of a home-based intervention targeted to parents with intellectual disability to promote child health and home safety in the preschool years. | Mixed methods RCT Measured: health comprehension, illness and symptom recognition, life threatening emergencies, going to the doctor, using medication safely. Ised the SF36 Personal Interview for subjective measure of parent health status. | Parents with intellectual disability |
| Lloyd & Coulson (2014) | United Kingdom | Journal Article | This research explored the experiences, perceptions, and clinical practices of learning disability nurses in order to identify specific barriers and facilitators influencing cervical screening utilisation by women with learning disabilities, and the role of learning disability nurses in promoting uptake. | Qualitative, semi-structured interviews | learning disability nurses |
| Marks & Sisirak (2014) | United States of America | Book Chapter | This chapter addresses the state of health promotion for people with intellectual disbaility in the USA. It gives an overview of factors contributing to health and wellness for individuals with intellectual disabilities and discusses the importance of developing comprehensive health promotion programmes that include health education, nutrition, and physical activity to improve the lives, self-determination, and community engagement of people with intellectual disabilities. | Literature review | People with intellctual disability |
| Marks et al. (2013) | United States of America | Journal Article | The primary aim of this research study was to evaluate the efficacy of a HealthyMatters Program: Train-the-Trainer Workshop on promoting and maintaining healthy behaviours of adults with ID. | Quantitative Randomised pre-test/post-test comparison (control) group design in CBOs of a staff-led health promotion programme. | Disability support workers People with intellectual disability |
| Marriot et al. (2014) | United Kingdom | Journal Article | This article describes our findings in relation to five English screening programmes, with particular reference to what specialist learning disability nurses, nurses working in screening services and primary care nurses can do to improve uptake of cancer screening by people with learning disabilities. | Literature review summarising the efforts of 5 English screening programs. | Health screening professionals People with intellectual disability |
| Martin et al. (2020) | Ireland | Journal Article | This paper sets out the background, approach, design and initial outcomes of a pilot health promotion initiative called Don't Mention the Diet! The initiative is a collaboration between three co-designers, an intellectual disability service provider and university that sought to address this issue of inaccessible health promotion information, which promotes the empowerment and self-determination of people with intellectual disability. |  | People with intellectual disability |
| Mastebroek et al. (2017) | Netherlands | Journal Article | This Delphi study aims to reach agreement between general practitioners (GPs), ID physicians and support workers on the principal actions and organisational factors facilitating HIE for people with ID who receive care from GPs and ID care provider services. We further aim to assess the perceived feasibility of these actions and factors in daily practice. | Mixed methods Modified two-round Delhi study. Design and distribution of a questionnaire on the organisational factores facilitating HIE. Open-ended response providing furher comments. | GPs Intellectual disability physicians Support workers |
| Mastebroek et al. (2016) | Netherlands | Journal Article | Our research question is: what are the barriers and facilitators experienced by people with ID and their (in)formal carers in the exchange of health information before, during and after completion of GP consultations? | Qualitative Semi-structured interviews and focus groups | People with ID Professional carers Relatives |
| McCormick et al. (2024) | United Kingdom | Journal Article | The purpose of this study was to identify the experiences of the Regional Hospital Passport from the perspective of the adult with intellectual disabilities, their families and health professionals following implementation. | Qualitative descriptive design | Adults with intellectual disabilities Their families Healthcare professionals |
| McIlfatrick et al. (2011) | United Kingdom | Journal Article | The overall aim of this study was to ascertain the healthcare professional’s perspective on their role in supporting women with intellectual disability to access breast cancer screening. | Qualitative Interviews and focus groups | Primary care staff and hospital breast screening staff |
| McPherson et al. (2017) | Australia | Journal Article | The aim of this study was to determine whether the school-based education/health inervention package, compared with usual care, increased the self-advocacy of adolescents with intellectual disability with regard to their health. | Quantitative (scale and category data) Parallel-group cluster RCT testing the perceived gains and effect on health advocacy. | Adolescents with intellectual disability Carers |
| Middleton et al. (2021) | United Kingdom | Journal Article | As part of intervention development, ahead of the LUSTRUM (Limiting Undetected Sexually Transmitted infections to RedUce Morbidity) partner notification randomised controlled trial of accelerated partner therapy (APT),7 we explored the perspectives of people with mild learning disabilities. We aimed to identify potentially modifiable elements of the partner STI/BBV self-sampling on August 13, 2024 by guest services research packs with the broader aim of increasing access to this type of self-managed care for people with mild learning disabilities and people with low health literacy more broadly. | Qualitative Interviews and focus groups with a semi-structured topic guide | Heterosexual people and men who have sex with men, with mild learning disabilities |
| Nair et al. (2023) | India | Journal Article | Our objective was to develop an evidence-based conceptual framework for a needs-based inclusive intervention through community consultations to reduce the risk of long-term health problems among children with ID in India. | Qualitative Community engagement and involvement activities using a community-based participatory approach | Parents of children with intellectual disability People who work with people with intellectual disability Policymakers, lawyerrs, NGOs Professionals from health, education, and social care |
| Nelson et al. (2020) | Sweden | Journal Article | Against this background, the overall aim of the present study was to explore what it meant for a group of teachers in southern Sweden to teach SRHR to students with intellectual disabilities | Qualitative Phenomenological approach - semi-structured interviews | Teachers of students with intellectual disability |
| Newman et al. (2023) | Australia | Journal Article | This qualitative research contributes new knowledge by using a health literacy framework to explore the research question: How is easy read and accessible information used by staff working with people with intellectual disability in mental health services. | Qualitative Semi-structured qualitative interviews | Mental health staff people with intellectual disability support people |
| Newman et al. (2022) | Australia | Journal Article | This paper examines the representation of the right to information for people with intellectual disability in Australian mental health policies. | Qualitative Content analysis of policy documents | People with intellectual disability |
| O'Leary et al. (2018) | United Kingdom | Journal Article | The aim of this study was to explore the organizational barriers and enablers to staff supporting people with intellectual disabilities to engage in regular physical activity and a healthy diet. | Qualitative Interviews and focus groups | Managers and frontline staff at residential services |
| Oosterveld-Vlug et al. (2021) | Netherlands | Journal Article | The aim of this study was to explore the difficulties Dutch people with intellectual disabilities experience during the process of considering and seeking medical help from their GP. | Qualitative Descriptive study, semi-structured individual interviews | People with intellectual disability Relatives |
| Overwijk et al. (2022) | Netherlands | Journal Article | The aim of this study is to evaluate the preparation, implementation, and preliminary outcomes of a theory-based training and education program for DSPs to learn how to support people with moderate to profound ID in a healthy lifestyle. | Mixed method | DSPs people with moderate to profound ID Managers/coorindators of the participating facilities Trainers of the programs |
| Overwijk et al. (2022) | Netherlands | Journal Article | To meet the need for theory-based interventions based on relevant determinants, the aim of this study is to develop a program for DSPs to support physical activity and healthy nutrition for people with moderate to profound levels of ID and to design its evaluation. | Descriptive paper | DSPs people with intellectual disability |
| Smith & Feinsinger (2024) | United States of America | Journal Article | This paper explores the possiblity of expanding the normative ideal of patient-centred communication to individuals with non-speaking intelelctual disabilities and asks what practices would enable clinicians to do so. | Extended essay | People with intellectual disability who are non-speaking |
| Pownall et al. (2016) | United Kingdom | Journal Article | An attempt was made to explore the impact of both intellectual ability and the extent of social networks on sexual health understanding. | Quantitative Health knowledge questionnaire Sources of information questionnaire Social network questionnaire WASI-II | People with ntellectual disability |
| Primeau & Talley (2019) | United States of America | Journal Article | The ID-COMMUNICATE model was developed to provide a flexible education model for nurses and other healthcare professionals on specialised communication techniques that are useful with adult patients who have communication, literacy, or cognitive issues. This article describes how the authors used the ID-COMMUNICATE educational framework to create a continuing education program to facilitate more sueccessful interactions between HCPs and patients. | Descriptive paper | HCP People with intellectual disability |
| Roll (2017) | United States of America | Journal Article | This article investigates the realities and the potential of health promotion for people with ID and has two goals. It analyses how health promotion is being conceptualised for people with ID in the literature and how health promotion can work best in the light of this group’s specific needs and limitations to improve their health and well-being. | Concept analysis | People with intellectual disability |
| Røstad-Tollefsen et al. (2020) | Norway | Journal Article | The aim of this study was to assess supporting staff's thoughts and experiences on factors influencing their opportunities to promote a healthy diet in adults with intellectual disability. | Mixed method Concept mapping: interviews, sorting, rating | Support networks |
| Pilskog Ruud et al. (2016) | Norway | Journal Article | The objective of this study is to identify factors that affect the caregivers' ability to promote a healhty diet among people with ID living in community residences in Norway. | Mixed method Concept analysis | Support networks |
| Sisirak & Marks (2014) | United States of America | Book Chapter | This chapter discusses strategies to support healthy food choices among people with intellectual disabilities using the Ecological Framework of Food Choice. | Lit review | People with intellectual disability |
| St John et al. (2021) | United States of America | Journal Article | The objective of this study is to understand how people with intellectual disability participate in and access health promotion. | Qualtiative Photovoice | People with intellectual disability |
| Strydom & Hall (2001) | United Kingdom | Journal Article | To test the hypothesis that medication information leaflets would imrpove the knowledge and care of the target group, the present authors designed an RCT and planned subgroup analyses where appropriate. | Quantitative Measured medication knowledge and satisfaction | People with intellectual disability |
| Sundblom et al. (2014) | Sweden | Journal Article | The aim of this study was to explore aspects important to the implementation process of this intervention, as perceived by health ambassadors and managers. | Qualitative Semi-structured interviews | Support workers |
| Taggart et al. (2011) | United Kingdom | Journal Article | The aim of this paper was to examine how communtiy nurses and residential staff support women with ID to access breast screening services. | Qualitative Focus groups | Community nurses Support workers |
| Temple & Walkley (2007) | Australia | Journal Article | This study explored factors perceived as enabling or inhibiting participation in physical activity by adults with ID from a health promotion perspective. | Qualitative Physical activity | People with intellectual disability Support workers Parents |
| Truesdale-Kennedy et al. (2010) | United Kingdom | Journal Article | This paper is a report of a descriptive study of understanding breast cancer experiences of breast mammography among women with an ID. | Qualitative Focus groups | People with intellectual disability |
| Umb Carlsson (2021) | Sweden | Journal Article | The aim of this study was to explore residents', staff members' and rehabilitation professionals' experiences of how a health-promotion intervention affected the habits of people living in a group home regarding eating habits and physical activities and staffs ways of working. | Qualitative Semi-structured focus groups | People with intellectual disability Support workers Rheabilitation professionals |
| van den Bemd et al. (2024) | Netherlands | Journal Article | This study aimed to explore the needs of patients with ID from the perspectives of both patients and HCPs in the context of chronic disease management in general practice. | Qualitative Interviews with PwID Focus groups with HCPs | People with intellectual disability HCPs |
| van Dooren et al. (2013) | Australia | Journal Article | This study aimed to identify the facilitators and barriers to registering for an eHealth record network for people with intellectual disability and those supporting them to manage their health information. | Qualitative Semi-structured interviews | People with intellectual disability Families Support workers |
| Vasudevan et al. (2024) | United States of America | Journal Article | In this qualitative study we explore the experiences and preferences of parents/guardians of AYA of childbearing potential with co-occuring epilepsy and ID regarding counseling by neurologists on SRH topics. | Qualitative Semi-structured interviews | Parents of parents/guardians of adults and young adults with ID |
| Vetter et al. (2022) | Germany | Journal Article | Based on interviews conducted within two research projects on the topics of health literacy and applying health information in people with intellectual disabilities with the corresponding target group, this article aims to build a secondary analysis that analyses the influence of the social context on health literacy of people with intellectual disabilities. | Qualitative Secondary analysis based on interviews conducted in two research projects. | People with intellectual disability |
| Vlot-van Anrooij et al. (2020) | Netherlands | Journal Article | This study aims to answer the following research question: "What assets for physical activity and healthy nutrition do people with moderate intellectual disabilities and proxy informations of people with severe/profound-intellectual disabilities identify and prioritise?" | Mixed methods Nominal Group Technique i) generating ideas ii) ranking | People with intellectual disability |
| Vlot-van Anrooij et al. (2019) | Netherlands | Journal Article | Our study aims to conceptualise healthy settings for people with ID. | Integrative mixed methods approach Concept mapping study: Brainstorming guided by focus prompts Sorting and rating of statements resulting from phase 1 | Researchers with experience in academic research and practice |
| Wahlstrom et al. (2014) | Sweden | Journal Article | The aim of this study was to explore aspects important to consider when promoting health amongst persons with intellectual disabilities in group homes, from the perspective of professionals. | Qualitative study Semi-structured interviews | Manager and caregiver of group homes |
| Willis et al. (2010) | United Kingdom | Journal Article | This study aimed to provide findings to complement those from a parallel study investigating the menopausal knowledge and experiences of 45 women with intellectual disabilities (Willis et al., in press). | Qualitative Semi-structured interviews | Women with intellectual disability Their carers |
| Wilson et al. (2018) | United Kingdom | Journal Article | The objective of this study was to evaluate and compare, quantitatively and qualitatively, the impact of two different educational interventions about testicular health on knowledge, skills and health-related behaviours in regards to self-reported TSE at 1 week and 6 months post-intervention in male adults with IDD. | Mixed methods Participatory randomized parallel study of two educational interventions Leaflet interventon Teaching intervention Measured: self-examination skills, health anxiety and self-efficacy, participant reported self-examination and GP visits over a six-month period, qualitative evaluation | Men with intellectual disability |
| Wiseman & Ferrie (2020) | United Kingdom | Journal Article | In this paper we argue that reproductive health inequalities, and their social determinants, contribute to this gendered inequality in health. We assert that socio-cultural assumptions, and legacies of psycho-emotional disablist practices, about the sexual citizenship of women with intellectual disabilities structure their exclusion from this sphere. | Qualitative Focus groups & open-answer questionnaires | Women with intellectual disability |

**Table S2**

| **Theme** | **Quality** | **Confidence** |
| --- | --- | --- |
| **People with intellectual disability**  Exclusion from information  Tailored and empowered approaches to information  Tailored delivery  Psychosocial processes  Trust, rapport, and autonomy in information exchange  Confidence and motivation  Independent and supported decision-making  Relevant information | 26 (B)  27 (B)  27 (B)  25 (B)  29 (A)  28 (B)  27 (B)  27 (B) | Moderate  Moderate  Moderate  Moderate  High  Moderate  Moderate  Moderate |
| **Support networks**  Facilitating access to information  Resources and education  Literacy mediation  Supporters are not medical experts  Upholding the right to disagree  Ethical dilemmas  Gatekeeping  Confidence in role | 26 (B)  23 (C)  28 (B)  26 (B)  26 (B)  27 (B)  28 (B)  27 (B) | Moderate  Low  Moderate  Moderate  Moderate  Moderate  Moderate  Moderate |
| **Healthcare professionals**  Source of information  Equipping mainstream professionals  Capacity to facilitate triadic communication  Rapport-building skills  Supporting choice | 26 (B)  28 (B)  28 (B)  32 (A)  24 (B) | Moderate  Moderate  Moderate  High  Moderate |
| **Social and environmental determinants**  Upholding the right to information and autonomy  Media as a source of information  Schools as a resource for health literacy | 27 (B)  28 (B)  28 (B) | Moderate  Moderate  Moderate |
| **Situational determinants**  Social and familial networks  Disability sector policies  Health sector policies | 25 (B)  27 (B)  29 (A) | Moderate  Moderate  High |
| **Personal determinants**  Intersectional identities  Intellectual disability is heterogenous | 27 (B)  28 (B) | Moderate  Moderate |
